# Assessing REALTER simulator: analysis of ocular movements in simulated low-vision conditions with extended reality technology

**DOI:** 10.3389/fbioe.2024.1285107

**Published:** 2024-04-04

**Authors:** Mattia Barbieri, Giulia A. Albanese, Andrea Merello, Marco Crepaldi, Walter Setti, Monica Gori, Andrea Canessa, Silvio P. Sabatini, Valentina Facchini, Giulio Sandini

**Affiliations:** ^1^ Department of Robotics, Brain and Cognitive Sciences, Istituto Italiano di Tecnologia, Genova, Italy; ^2^ Department of Informatics, Bioengineering, Robotics and Systems Engineering, University of Genoa, Genoa, Italy; ^3^ Electronic Design Laboratory, Istituto Italiano di Tecnologia, Genova, Italy; ^4^ Unit for Visually Impaired People, Istituto Italiano di Tecnologia, Genova, Italy; ^5^ Fondazione David Chiossone per Ciechi e Ipovedenti, Genoa, Italy

**Keywords:** extended reality, augmented reality, rehabilitation, visual impairments, eye tracking, immersive technology, gaze-contingency, ocular movements detection

## Abstract

Immersive technology, such as extended reality, holds promise as a tool for educating ophthalmologists about the effects of low vision and for enhancing visual rehabilitation protocols. However, immersive simulators have not been evaluated for their ability to induce changes in the oculomotor system, which is crucial for understanding the visual experiences of visually impaired individuals. This study aimed to assess the REALTER (Wearable Egocentric Altered Reality Simulator) system’s capacity to induce specific alterations in healthy individuals’ oculomotor systems under simulated low-vision conditions. We examined task performance, eye movements, and head movements in healthy participants across various simulated scenarios. Our findings suggest that REALTER can effectively elicit behaviors in healthy individuals resembling those observed in individuals with low vision. Participants with simulated binocular maculopathy demonstrated unstable fixations and a high frequency of wide saccades. Individuals with simulated homonymous hemianopsia showed a tendency to maintain a fixed head position while executing wide saccades to survey their surroundings. Simulation of tubular vision resulted in a significant reduction in saccade amplitudes. REALTER holds promise as both a training tool for ophthalmologists and a research instrument for studying low vision conditions. The simulator has the potential to enhance ophthalmologists’ comprehension of the limitations imposed by visual disabilities, thereby facilitating the development of new rehabilitation protocols.

## 1 Introduction

More than 285 million people worldwide suffer from visual impairments (VIs) (Gori et al., 2016). Among them, 39 million are completely blind, while 10 million experience chronic VIs that affect a partial portion of their field of view (FOV), known as low vision diseases, such as glaucoma, hemianopsia, and age-related macular degeneration (Flaxman et al., 2017). These chronic Vis cannot be corrected with glasses, contact lenses, or surgery and significantly impact daily activities (Leat et al., 1999). Typically, individuals affected by low vision diseases experience a significant loss of their visual field, affecting the central (maculopathy), peripheral (tunnel vision), or half of the vertical visual field (hemianopsias). These conditions impair activities such as reading (Rubin, 2013), driving, recognizing objects (Cahill et al., 2005), and faces (Taylor et al., 2016). Additionally, visual field distortions significantly compromise the oculomotor system, resulting in eye movement anomalies, especially in saccades (Nuthmann et al., 2022). The form of VIs and their resulting oculomotor instabilities differ according to specific pathologies.

Regarding maculopathies, the National Eye Institute (Rickman et al., 2013) outlined four main stages associated with their symptoms. In the early stage, individuals may exhibit minimal symptoms except for slight blurring of the image in the central visual field. In the intermediate stage, central visual field obstruction by scotoma leads to blurred vision and metamorphopsia, where straight lines appear wavy or crooked start to appear. In the advanced stage, blurred vision and metamorphopsia are compounded by the presence of gray-dark spots. In the most severe cases, scotomas manifest as image distortion and blurred effects overlaid with overlapping gray-dark spots (Hubschman et al., 2009). People with disabilities correlated with binocular maculopathy, frequently encounter difficulties with tasks requiring a central visual field (Cahill et al., 2005). Maculopathy significantly affects the oculomotor system (Kumar and Chung, 2014). While saccades in healthy individuals are generally horizontally linear in magnitude, maculopathy induces multiple short saccades in various directions (Vullings and Verghese, 2021). Especially during the reading of a text, poor oculomotor control induced by central scotomas leads to abnormalities in interword saccades and an increased number of regressive saccades (Cahill et al., 2005).

The term “hemianopsia” refers to a group of binocular VIs affecting half of the visual field, typically one side of the vertical midline. Impaired hemifields are often obstructed by blurred vision and gray spots in the peripheral areas, rendering those portions of the visual field effectively blind (Wolberg et al., 2022). Hemianopsia can be divided into two main types. First, the heteronymous hemianopsia that encompasses bitemporal and binasal hemianopsia, affecting different areas of the visual field in each eye (Cushing and Brain, 1925; Igersheimer, 1947). Secondly, the homonymous hemianopsia which affects the same vertical midline in both eyes, resulting in the loss of a complete hemifield bilaterally, including half of the macula. Eye movement patterns are disrupted, with more frequent shorter saccades (especially regressive saccades) toward the blind field, and longer fixations in terms of durations (Zihl 1995). The loss of the left half of the vertical visual field affects reading performance, both by causing difficulties in finding the next line in a text (Zihl 1995) and by inducing several errors in the essential information needed to identify a word contained in its initial letters (Grunda et al., 2013).

The last disease under consideration is the loss of peripheral vision (i.e., tubular vision) that leads to a tunnel-like circular visual field. This condition obstructs daily activities, particularly spatial navigation and object recognition (Ophthalmology, 1960). Tubular vision presents initially as a blurred effect in the superior or inferior visual field, progressing to tunnel vision characterized by blurred effects, lack of information, and dark gray peripheral scotomas (Crabb et al., 2013; Gupta et al., 2016). Fixation instabilities and altered saccades are common in individuals with tubular vision, affecting daily tasks such as reading (Nguyen et al., 2014; McDonald et al., 2022).

Many rehabilitation procedures such as prismatic correction (Moss et al., 2014) and compensatory training (Mannan et al., 2010) have been implemented to date. Nonetheless, the World Health Organization reported the need for qualified rehabilitation professionals to apply new user-centered and trans-disciplinary approaches in daily practice (WHO, 2019). The first step of this process would be to train rehabilitators with tools that simulate low-vision diseases to understand how low-vision individuals perceive and interact with the external environment. In particular, by perceiving and navigating the world as low-vision individuals, ophthalmologists and researchers might improve the current scenario of VIs rehabilitation by discovering new rehabilitation techniques.

The question that has emerged in the field is: what is a proper method to exploit the benefit of simulations? Immersive technologies have been demonstrated to represent noteworthy devices to simulate realistic scenarios thanks to their capability to elicit a sense of immersion (Suh and Prophet, 2018) and presence (Thornson et al., 2009). Specifically, Soliman et al. (2017) defined immersive technologies as tools that blur the line between the physical and virtual world creating a sense of immersion and enhancing the realism of the virtual experience. Nowadays, the term “immersive technologies” is used to refer to several different technologies, such as virtual reality (VR), augmented reality (AR), spatial audio, and haptic technologies (Handa et al., 2012). It is common to use the term “extended reality” (XR) to indicate a macro category that includes both VR and AR, where VR implies users’ immersion entirely in a digital-simulated world (Greengard, 2019), whereas AR combines the digital and physical world, allowing the aligned overlap of 3D digital objects in the real world (Craig, 2013). The peculiarity of exploring the world—real or simulated -, in 3 degrees of freedom (sitting position) or 6 degrees of freedom (standing position) typical of immersive XR, allowed the overcoming of the limitations of static 3D displays. These technologies have already shown their potential in healthcare and rehabilitation for people presenting with attention deficit hyperactivity disorder (Adams et al., 2009), and post-traumatic stress disorder (Kothgassner et al., 2019). Moreover, the use of immersive technologies was demonstrated to enhance learning experiences (T. C. Huang et al., 2016), and foster participation (Fonseca et al., 2014; M. Huang et al., 2010).

A groundbreaking study by Jones et al. (2020), demonstrated the effectiveness of gaze-contingent simulations in both VR and AR through multiple HMDs for accurately simulating VIs in healthy-sighted subjects. Additionally, Chow-Wing-Bom et al. (2020) researched to assess the impact of peripheral vision diseases on daily life activities. Twelve healthy-sighted subjects explored a virtual environment in VR, where the levels of peripheral vision loss were independently manipulated in each eye for each trial. The data revealed that even with significant visual impairment, subjects could successfully complete tasks.

Along this line, we recently introduced the REALTER (wearable egocentric altered reality simulator) project (Barbieri et al., 2023). REALTER is an immersive simulator capable of altering normal sight in real-time to simulate various low-vision conditions using an HMD in a real environment (see Supplementary Video S1 for a demonstration). The Chiossone Foundation for Blind and Low-Vision Individuals in Genoa collaborated on this project, which is part of the European oMERO project (Casiddu et al., 2023), aimed at evaluating the effectiveness of low-vision simulations. REALTER utilizes AR with a video see-through (VST) paradigm, allowing real-world vision mediated by external cameras attached to the HMD. By identifying common diseases requiring rehabilitation, we designed realistic gaze-contingent simulations for various low vision conditions, including loss of central and peripheral vision, hemianopsia, and additional VIs such as achromatopsia, cataracts, and diabetic retinopathy. Considering the peculiarity of the system not to augment, but to alter the reality to recreate low vision individuals’ perspective, we used the term *alTered Reality* (TR) instead of AR to define our approach.

The primary goal of the REALTER project is to provide visual rehabilitation centers with a validated tool to assist ophthalmologists in understanding visually impaired individuals’ experiences and developing tailored rehabilitation protocols. Our study aimed to validate TR by collecting data from healthy-sighted subjects performing daily actions under simulated low-vision conditions, such as reading, pouring water, and interacting with objects. The realism of the simulations led us to hypothesize that TR could induce alterations in healthy individuals’ oculomotor behavior similar to those caused by maculopathy, hemianopsia, and tubular vision. Our experimental protocol considered three different low-vision conditions: advanced binocular maculopathy, homonymous hemianopsia, and tubular vision. The system enabled the analysis of subjects’ ocular movements across these conditions, providing insights into compensation strategies for different types of VIs.

Our results indicate that the system is capable of producing distortions in eye movements that vary across the three disabilities and normal vision. Additionally, these distortions resemble the behavior of healthy-sighted individuals and those with low vision in non-simulated conditions (Goodwin, 2014; Nguyen et al., 2014; Verghese et al., 2021; McDonald et al., 2022) suggesting that TR is capable of realistically simulating normal and impaired visual conditions and potentially inducing alterations in ocular movements similar to those observed in individuals with maculopathy, hemianopsia, and tubular vision.

## 2 Methods and analysis

### 2.1 Participants

Participants were 18 healthy-sighted adults with a mean age of 28 years, with no experience in low-vision rehabilitation. We defined healthy sight as monocular letter acuity ≤0.3 logMAR. Additionally, subjects have not declared to suffer from VIs. Subjects gave their informed consent to participate in the study. The study was approved by the ethics committee of the local health service (Comitato Etico, ASL 3, Genova, Italy). All experiments were conducted in accordance with the Declaration of PP, (1964). Informed written consent was obtained for all the subjects.

### 2.2 Immersive system design

The altered reality (TR) is displayed through a VST HMD with an integrated eye tracker (HTC Vive Pro Eye), augmented with external cameras (Stereolabs Zed Mini) able to acquire images from the real world. HTC Vive Pro Eye has a resolution of 2,880 × 1,600 pixels (1,440 × 1,600 per eye), with a 110° FOV and a refresh rate of 90 Hz. It integrates a Tobi eye tracker which detains an accuracy (within FOV 20) of 0.5°–1.1° with a sampling frequency (binocular) of 120 Hz. In addition, the HTC Vive Pro Eye requires two external base stations to trigger SteamVR Tracking, a G-sensor, a gyroscope, and a proximity sensor. Stereolabs Zed Mini has been anchored to the headset to enhance pass-through resolution; the camera has a resolution of 720p and a FOV of 90° (horizontal) and 60° (vertical). The device is wired to a backpack PC (HP VR Backpack G2) which is equipped with an Intel Core i7-8850H processor and NVIDIA GeForce RTX 2080 graphic card with 8 GB dedicated GDDR6. The HTC Vive Pro Eye as a tool for measuring saccades in VR was already exploited by Imaoka et al. (2020) with positive results.

The software is written in Unity C# (version 2021.3.3f1). The current version of the software provides an exhaustive portfolio of low vision conditions able to offer different stages of maculopathy, hemianopsias, and tubular vision. Every element of the system is called in the motor engine through multiple software development kits (SDK): SteamVR v1.14.15 (for VR headset), ZED SDK v3.7 (for external AR pass-through cameras), and SRanipal SDK v1.3.6 (for eye tracking). The pass-through camera required the installation of ZED SDK 3.7.0, Cuda 11.6.1,SR Runtime, SteamVR 2.7.3.

**FIGURE 1:**
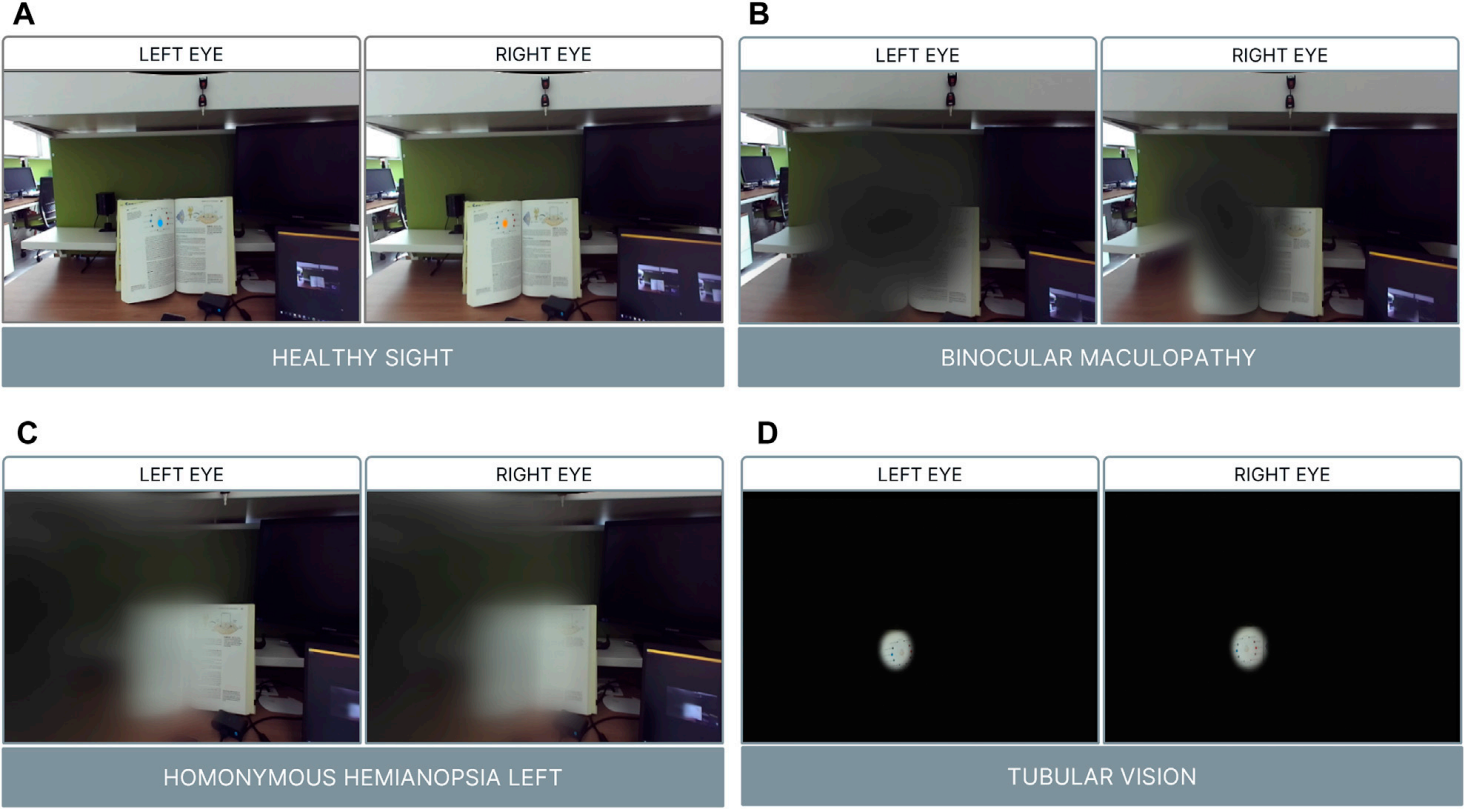
Visual conditions. Panel **(A)** Healthy sight with fixations points for each eye; Panel **(B)** Binocular Maculopathy; Panel **(C)** Homonymous Hemianopsia Left; Panel **(D)** Tubular Vision.

The integrated eye tracker allows the recording of data about subjects’ ocular movements and the design of gaze-contingent simulations. To conduct quantitative analysis, we used both eyes and head data. HTC Vive Pro Eye provides data from both the left and right eye through SRanipal SDK: validity of eye data, eyes openness, pupil diameter (mm), pupil position (normalized vector between 0 and 1), gaze origin (mm), and gaze direction (normalized vector between −1 and 1). Data from both eyes were extracted from VerboseData in the struct data of ViveSR.anipal.Eye.EyeDatav2. Head data were extracted from Unity.Engine.XR in the struct data of InputTracking.GetLocalPosition(XRNode.CenterEye). We used time information from Unity.Engine in the form of Time.fixedTime. Because most low-vision diseases affect the same part of the retina while the eyes are moving, a gaze-contingent paradigm is required to update the position of the disability according to the subject’s fixation point. The term gaze indicates the combination between the head and eye movements (Holmqvist et al., 2011a). The gaze-contingent paradigm is an interactive eye tracker application used in computer science to refer to the ability of a computer screen to change in function depending on the viewer’s fixation point (Duchowski Theory, 2017). Therefore, the implementation of gaze-contingency required the combination of eyes and head movements. By combining gaze information from SRanipal and head movements from SteamVR, it was possible to integrate the gaze-contingent paradigm (Figure 1A). Specifically, we used the Unity function Transform.Direction to process data related to eye movements in conjunction with information about head movements. The position of the area of impaired vision was regulated by the resulting contingent vector that combined data from both sources to understand the gaze direction.

Low-vision diseases were replicated in TR by overlapping 2D images and 3D Unity’s game object rendered with shaders. These simulations were overlaid onto real-world scenes captured by Zed Mini cameras. Different techniques were employed for each visual condition. For simulating binocular maculopathy (Figure 1B), a 2D image of a dark gray spot was used to obscure the central visual field. Additionally, a shader applied to a 3D object implemented a warping effect to mimic the distortion caused by metamorphopsia. This effect was achieved by altering individual pixels with shifted texture samples, introducing non-stationarity through modulation of offset values with a periodic temporal signal, and encoding these offsets in a gaze-contingent deformation map generated by summing a simulated force field. In the case of tunnel vision (Figure 1D) simulation, a 2D black image with a central hole was overlaid, along with a shader producing a dark-blurred effect. This effect allowed the subject to maintain clear vision in the center of the FOV while obstructing the peripheral vision. Lastly, homonymous hemianopsia on the left side (Figure 1C) was simulated using a 3D plane rendered with a shader to create a blurred effect on the left side of the vertical FOV. A dark-blurred effect was applied in both tunnel vision and hemianopsia simulations to reduce visual acuity using Gaussian blur, a multiresolution pyramid approach, and a depth-of-field effect. All three simulations utilized the gaze-contingent paradigm, with experimenters using the PC keyboard to trigger low vision disabilities and control data storage.

Each low vision simulation was qualitatively evaluated by expert ophthalmologists from the Chiossone Foundation in specific evaluation phases to assess their realism.

### 2.3 Experimental protocol: overview

The goal of the experiment was the validation of REALTER by performing four daily activities: reading, reading, filling a glass of water and interacting with specific objects. Each task was performed under four different simulated visual conditions: binocular maculopathy, tubular vision, homonymous hemianopsia, and healthy sight.

In agreement with the ophthalmologists of the Chiossone Foundation, we focused on the worst case of each family of VIs stored in the simulator portfolio. In particular, the simulator focused on replicating three specific low vision conditions: *i*) an advanced form of maculopathy, corresponding to severe cases of the condition *ii*) homonymous hemianopsia affecting the left hemisphere, which is known to cause difficulties in identifying words based on initial letters in a text *iii*) an advanced stage of glaucoma characterized by VIs in the peripheral field, rendering the acquisition of any information challenging. These conditions were selected based on input from ophthalmologists, who identified them as particularly impactful on the daily lives of affected individuals.

The simulations were presented in a random order to prevent learning effects, with the presentation of normal vision always occurring last. The objective was to evaluate the impact of different low vision conditions on task performance. Performance was assessed based on factors such as execution time, as well as the number and magnitude of saccadic eye movements, and the duration of fixations. During each task, participants were seated in front of a table. We opted to allow free movement and rotation of the head for two main reasons: firstly, keeping the head fixed would have compromised the realism of the experimental tasks, and secondly, it could have restricted participants in their exploration of compensatory techniques. Each task was carefully selected in collaboration with ophthalmologists from the Chiossone Institute in Genoa. We prioritized tasks that mimic everyday activities, such as reading text or pouring from a bottle, which individuals with low vision often require assistance with. Our aim was to discern differences in eye and head movements among the simulated VIs.

### 2.4 Experimental protocol: calibration process and familiarization

**FIGURE 2:**
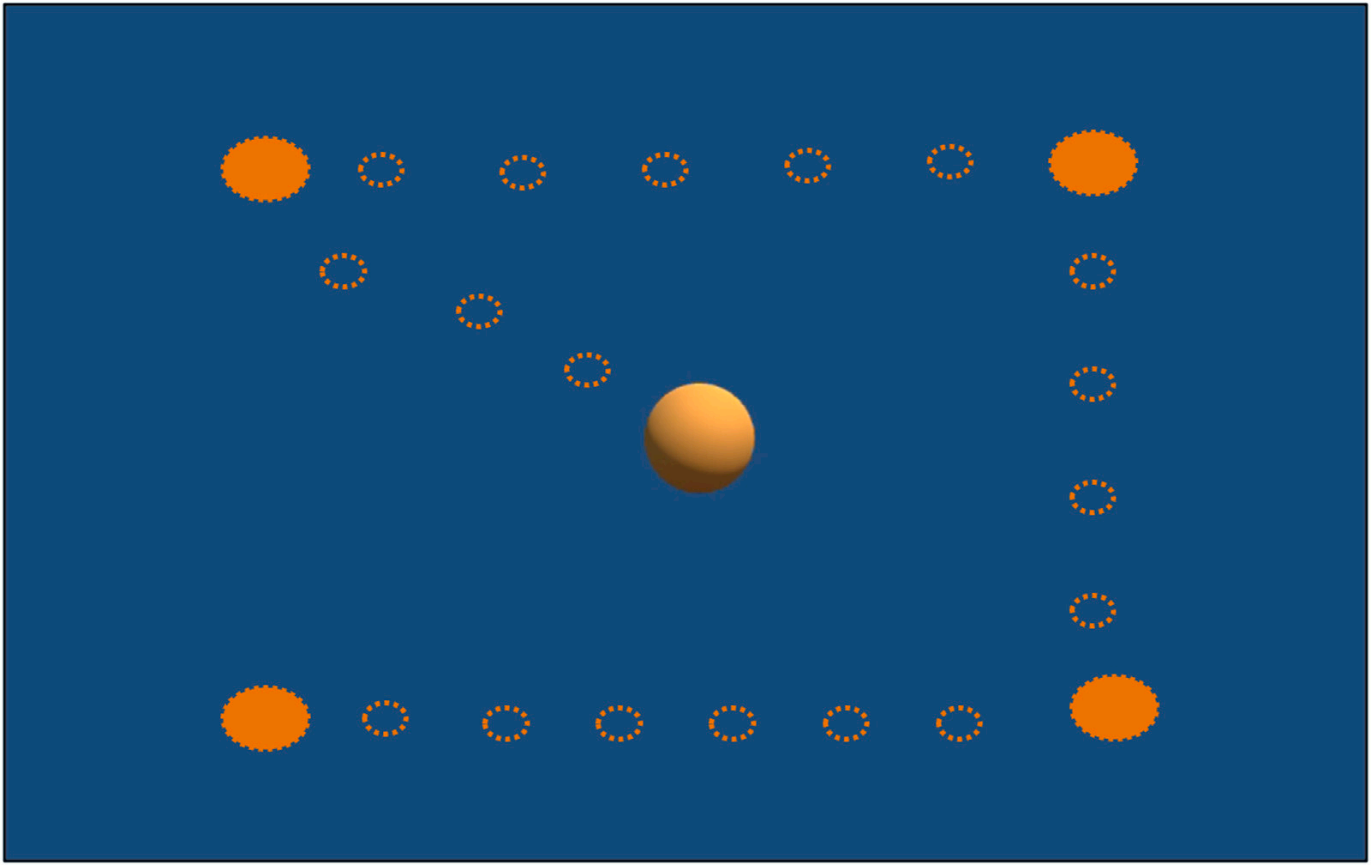
Calibration custom task: Subjects should follow with their eyes the sphere’s movements: the empty circles show the sphere’s path; the full circles show the positions where the sphere remained fixed for 3 s.

**FIGURE 3:**
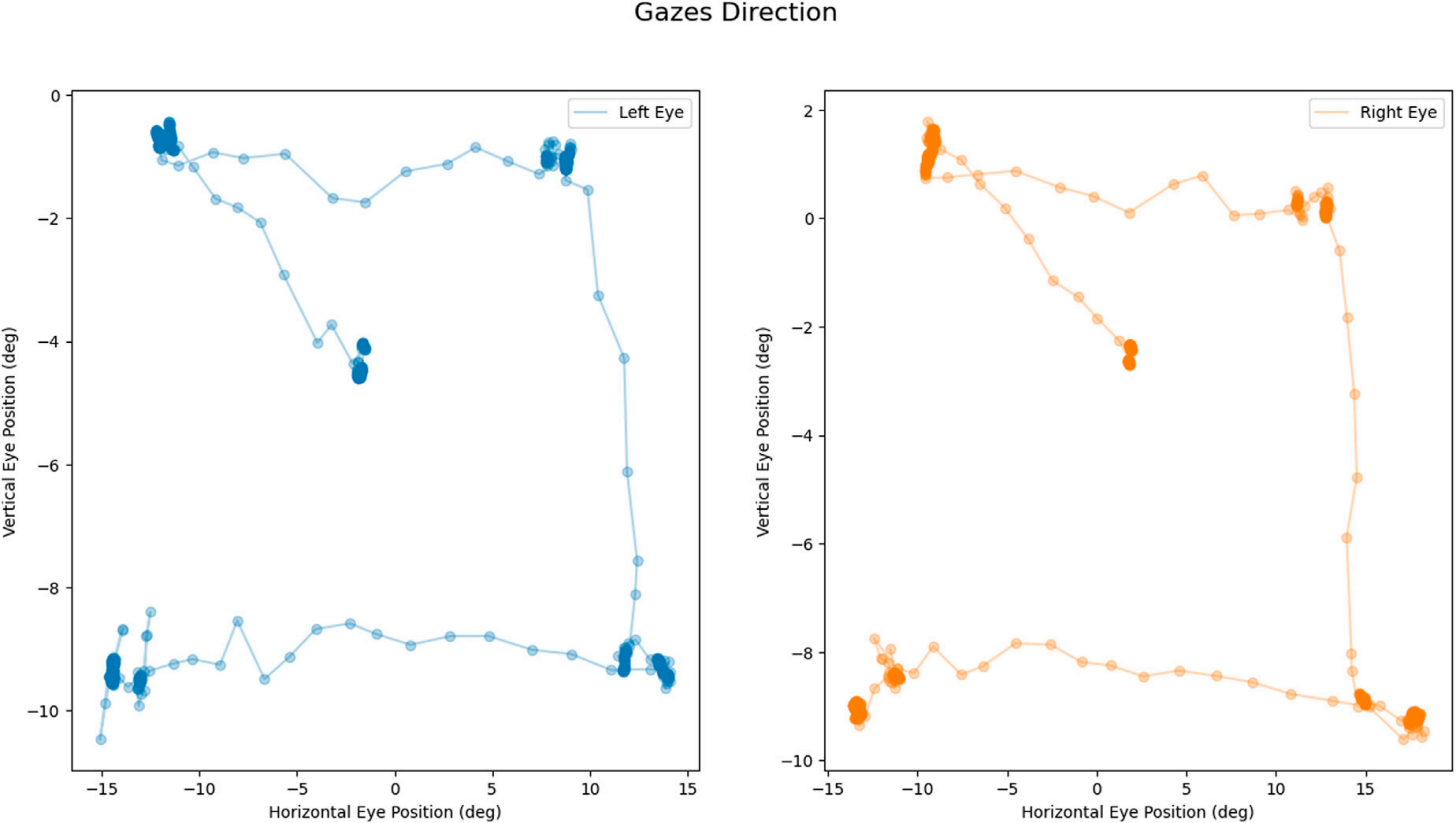
Calibration task output: Example of coherent scan paths during the task: left eye movement in blue (on the left); right eye movement in orange (on the right). All measures are expressed in degrees.

REALTER required appropriate calibration for each subject. The HTC Vive Pro Eye provided a standard calibration procedure, involving manual adjustment of the head-mounted display (HMD) position to ensure proper interpupillary distance between the eye lenses and calibration of the eye tracker through a 5-point calibration routine. At the outset of the experimental session, a custom saccade detection task was incorporated to verify the accuracy of the eye tracker data. In this task, participants were instructed to track the movement of a sphere within a virtual environment as the sphere changed position four times (see Figure 2). Subsequently, the experimenter conducted a brief analysis by plotting the eye movements’ scan path. If the scan path aligned with the movement of the sphere (as depicted in Figure 3), the data were deemed valid. Conversely, if the scan path did not match the sphere’s movement, the calibration process had to be restarted from the beginning. To familiarize subjects with the simulated low vision conditions and enhance their comfort, a familiarization session was conducted prior to the tasks. During this phase, participants engaged in simple actions such as scanning the environment and interacting with objects under the three different low-vision conditions.

### 2.5 Experimental protocol: reading

**FIGURE 4:**
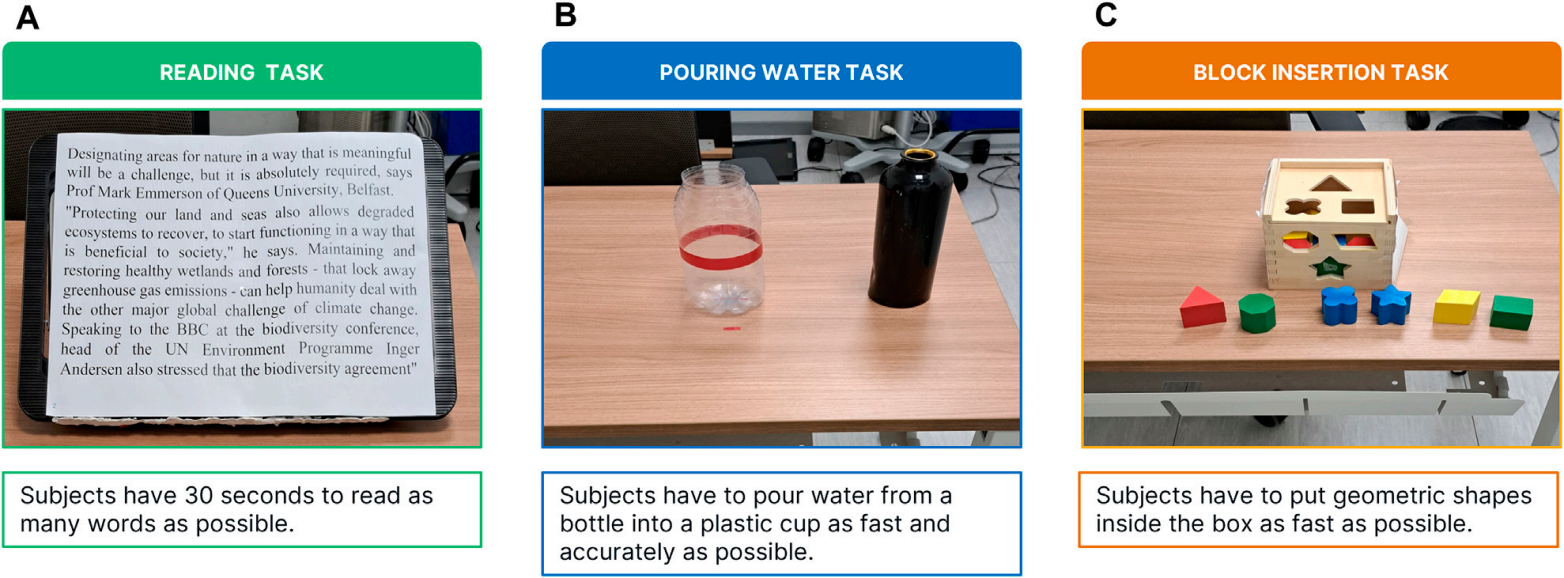
Experimental objects: Panel **(A)** A3 paper for the reading task; Panel **(B)** cup and bottle used in the pouring water task; Panel **(C)** the shape-sorting classic cube toy and geometric shapes used in the block insertion task.

During the reading task, subjects were asked to read a text written in their native language on an A3 format paper board for a duration of 30 s (see Figure 4A). The font size of the text was 17.5 mm. The paper board was positioned on a stand in front of the subjects, fixed at a distance of 40 cm from their eyes, following the protocol of the MNREAD Acuity Chart test (Legge et al., 1989). Participants were instructed not to interact with the board and to maintain a fixed distance of 40 cm from the stand. They were directed to read as many words as possible within the given time frame. The length of the text ensured that subjects would not finish reading the entire paper page within the allotted time. Different paper pages were used for each visual condition. The outcome measures included the number and magnitude of saccadic eye movements and the number of words read.

### 2.6 Experimental protocol: pouring water

During the Pouring Water task, participants were required to pour water from a 1-L bottle into an empty cylindrical plastic cup, with dimensions of 7.8 cm in diameter and 24 cm in height. The cup was positioned in front of the subject, 50 cm away (refer to Figure 4B). While executing the task, the subject was allowed to touch the cup but not move it. The full bottle was placed either 15 cm to the right or left of the cup, depending on the subject’s dominant hand. Participants were instructed to pour the water with both accuracy and speed, aiming to fill the cup up to a red stripe indicated on its surface. The task concluded when subjects verbally declared that they had reached the red stripe. Outcome measures included the assessment of the number and magnitude of saccadic eye movements and fixations, as well as the duration of the task.

### 2.7 Experimental protocol: block insertion

The task involved solving a classic shape-sorting cube toy (see Figure 4C). The cube, with dimensions of 14 × 14 × 14 cm, was positioned 50 cm in front of the subjects, who were only permitted to interact with the top and front faces of the cube (the left and right faces were obscured). Participants were tasked with inserting six geometric shapes, located between themselves and the cube, into their respective cavities on the cube’s surface. Subjects were given the freedom to select the order in which they inserted the shapes, but they were not allowed to manipulate the cube itself. The task concluded once the subject had successfully inserted all six shapes into their corresponding cavities. To prevent learning effects, the cube was rotated in each condition to present a different face, along with corresponding geometric shapes for insertion. Outcome measures included the assessment of the number and magnitude of saccadic eye movements, as well as the duration of the task.

### 2.8 Data processing and analysis

**FIGURE 5:**
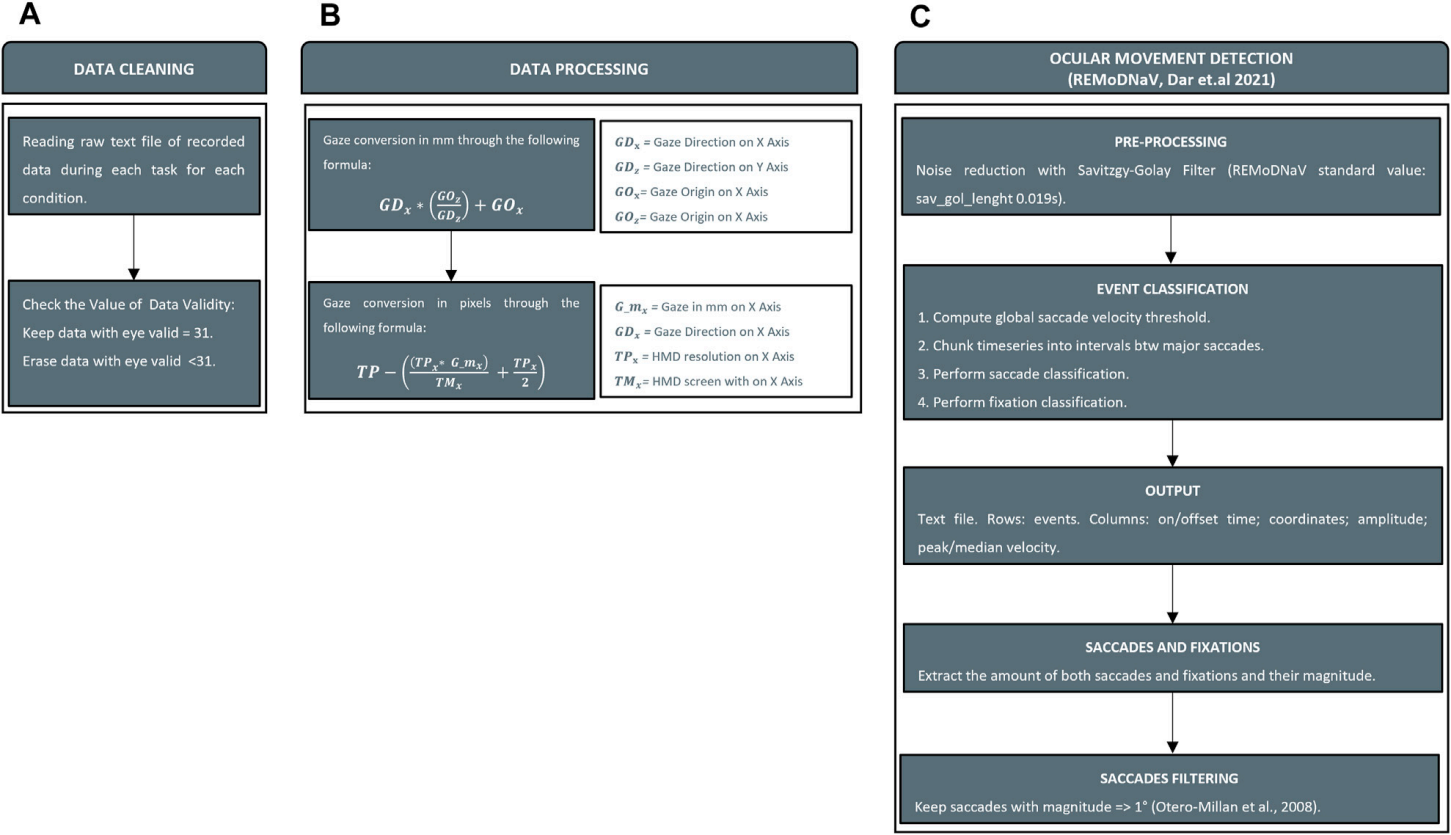
Data processing. Panel **(A)** Cleaning invalid value from data recorded. Panel **(B)** Conversion of gaze information from raw data (gaze origin and gaze direction) in mm and pixels. Panel **(C)** Ocular movements detection with REMoDNaV and saccades filtering.

Thanks to the embedded sensors and eye-tracking technology, we recorded ocular movements and head kinematics to extract quantitative outcomes for the analysis. To identify the nature of ocular movements, we employed an existing software written in Python language (REMoDNaV) (Dar et al., 2021) able to analyze eye tracking data listed above. REMoDNaV is built on the algorithm by Nystrom and Holmqvist (Nyström and Holmqvist, 2010) that employs an adaptive approach to velocity-based eye-movement event classification (Dar et al., 2021). The software was successfully implemented in multiple studies inherent to the analysts of eye data from XR HMD (Liebers et al., 2021). In its current state, REALTER is designed to measure saccades and fixations. Despite having a sampling frequency of around 120 Hz, the Tobii eye tracker integrated into the HTC Vive Pro eye is defined as low speed. This characteristic leads to several limitations in eye tracking, such as the inability to detect fixational micromovements like microsaccades, drifts, and tremors (Holmqvist et al., 2011b). As a consequence, the eye tracker detected those micromovements as periods of time during which the eyes were kept almost still and the data analysis by REMoDNaV categorized them as fixations (velocity lower than 2°/s). Nevertheless, low-speed eye trackers (with sampling frequency <250 Hz) are currently employed in research fields to collect data (Gibaldi and Sabatini, 2021). Initially, data were cleaned by removing all data with an eye validity of less than 31 as indicated by Imaoka et al. (2020) (Figure 5A). Secondly, since REMoDNaV required input eye data in pixels, we converted gaze in mm (Eq. 1) and then in pixels (Eq. 2) by considering gaze origin and gaze direction provided by SRanipal (Figure 5B). To find the position of the eye on the screen in pixels, Eqs 1, 2 were used. Specifically, Eq. 1 gives the position on the horizontal (x) axis in mm, which is then converted to pixels using Eq. 2. The same procedure was used to find the position on the vertical (y) axis in mm and pixels.

$${G\_m}_{x}={GD}_{x}*\left(\frac{{GO}_{z}}{{GD}_{z}}\right)+{GO}_{x}$$ (1)

${GD}_{x}$ : Normalized Gaze Direction on X-Axis

${GD}_{z}$ : Normalized Gaze Direction on Z-Axis

${GO}_{x}$ : Gaze Origin in mm on X-Axis

${GO}_{z}$ : Gaze Origin in mm on Z-Axis

$${G\_p}_{x}=TP-\left(\frac{\left({TP}_{x}*\;{\;G\_m}_{x}\right)}{{TM}_{x}}+\frac{{TP}_{x}}{2}\right)$$ (2)

${\;G\_m}_{x}$ : Gaze Direction in mm on X-Axis

${GD}_{x}$ : Normalized Gaze Direction on X-Axis

${TP}_{x}$ : HMD resolution on Horizontal Axis (2,880 pixels)

${TM}_{x}$ : HMD screen width on Horizontal Axis (119 mm)

The second stage involved the interpretation of ocular movements with REMoDNaV (Figure 5C). Although SRanipal stored data related to each eye, the analysis of ocular movements was performed on each subject’s dominant eye assessed using the hole-in-the-card test (Yang et al., 2010). As output of the analysis, REMoDNaV identified ocular movements. In particular, we focused on the number and amplitude of saccades and the duration of fixations. REMoDNaV detects saccadic eye movements with no distinction between saccades and micro-saccades. In order to clean counting from all putative micro-saccades, we removed all saccades with a magnitude of less than 1° as reported in a study on this topic (Otero-Millan et al., 2008).

Subjects’ performance was evaluated in terms of the number of words read in the reading task, and the time to complete the task in the water and shapes tasks. For each task and condition, fixations were evaluated in term of durations (ms), and saccades in terms of amplitude (°) and rate of occurrence (event/s). The latter was calculated by dividing the number of saccades performed by the time taken to perform the task. Head rotations (HR) were evaluated by considering the total rotational shift performed during each task in each condition (Eq. 3). These metrics allow us to compare binocular maculopathy, homonymous hemianopsia, tubular vision, and healthy sight in terms of ocular movements, head movements, and task performance. As the data were not normally distributed, the vision conditions were compared using Friedman’s Anova. When significant (*p* < 0.050), post-hoc Tukey-corrected pairwise comparisons were performed to determine which conditions showed significant differences.

$$HR:\sum_{i=1}^{n-1}\sqrt{{\left(a_{i}-\;a_{i+1}\right)}^{2}+{\left(\beta_{i}-\;\beta_{i+1}\right)}^{2}+{\left(\gamma_{i}-\;\gamma_{i+1}\right)}^{2}}$$ (3)

a : Rotation about X-Axis;

β : Rotation about Y-Axis;

γ : Rotation about Z-Axis;

i : i-th time sample;

N : Task entire duration.

## 3 Results

Each task has been evaluated in terms of task performance (words read or time to perform) and ocular movements. In the next sections, low-vision disabilities are abbreviated as B (binocular maculopathy), H (homonymous hemianopsia on left hemifield), and T (tubular vision), while the healthy sight condition is denoted as N (normal vision).

### 3.1 Ocular movements assessments in healthy sight

First, we evaluated ocular movements performed by subjects wearing the HMD in the healthy sight condition (N): we assessed saccadic eye movements for median magnitude and fixations for median durations.

• reading task: (saccades amplitude: ∼7°; fixations duration: ∼135 ms);
• water pouring task: (saccades amplitude; fixations duration: ∼230 ms);
• block insertion task: (saccades amplitude ∼6°; fixations duration: ∼160 ms).

### 3.2 Reading task

**FIGURE 6:**
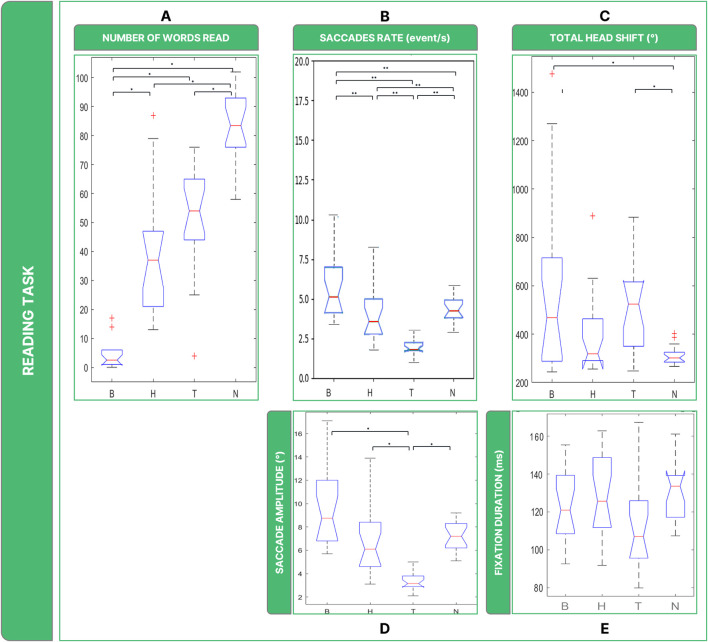
Boxplots of the results of the Reading Task: Panel **(A)** number of words read in 30 s; Panel **(B)** saccades rate; Panel **(C)** total head movements. Panel **(D)** saccades magnitude. Panel **(E)** fixations durations. Legend: (*: *p*-value <= 0.050).

Investigating subjects’ performance in terms of words read (Figure 6A), Friedman’s Anova revealed significant differences between groups ( $X^{2}$ = 46.9, *p* < 0.001). B was the most invalidating low vision condition (on average less than 5 words read), with *post hoc* tests revealing that the number of words read by subjects in B was significantly lower than in the other conditions (B vs. H: *p* = 0.001; B vs. T: *p* = 0.001). Subjects in the healthy sight condition read the highest number of words (∼85 words; B vs. N: *p* = 0.001; H vs. N: *p* = 0.002; T vs. N: *p* = 0.002). H and T shared common features, with an average of 38 and 45 words read respectively, and no significant differences were found (*p* = 0.315).

Statistically, the rate of saccades ( $X^{2}$ = 37.4, *p* < 0.001) (Figure 6B) was significantly different between B, H, and T (B vs. H: *p* = 0.012; B vs. T: *p* = 0.006; T vs. H: *p* = 0.006) and they behaved differently than N (B vs. N: *p* < 0.036; T vs. N: *p* = 0.006), with the exception of H which did not show statistical difference (H vs. N: *p* < 0.852).

The analysis of ocular movements showed that all low vision conditions shared comparable results in terms of saccades. Regarding the amplitude in degrees of the ocular movements, there are considerable differences between low vision conditions for saccades (Figure 6D) ( $X^{2}$ = 37.4, *p* < 0.001). The amplitude of saccades was particularly low in T (∼4°), with *post hoc* tests revealing significantly higher values than in the other two low vision conditions (B vs. T: *p* < 0.001; H vs. T: *p* < 0.001) and N (T vs. N: *p* = 0.001). Although the Friedmann Anova resulted slightly significant ( $X^{2}$ = 7.087, *p* < 0.049) (Figure 6E), *post hoc* test did not reveal significant differences in fixation duration among all conditions considered in this study.

It is also possible to highline significant differences between conditions when considering overall rotations performed by the head ( $X^{2}$ = 13.3, *p* < 0.004) (Figure 6C). Specifically, the post-hoc test revealed that in the B and T conditions the amount of head rotations performed by the subjects was comparable (B vs. T: *p* = 1), and both B and T induced them to rotate more than in N (B vs. N: *p* = 0.004; T vs. N: *p* = 0.001).

### 3.3 Pouring water task

**FIGURE 7:**
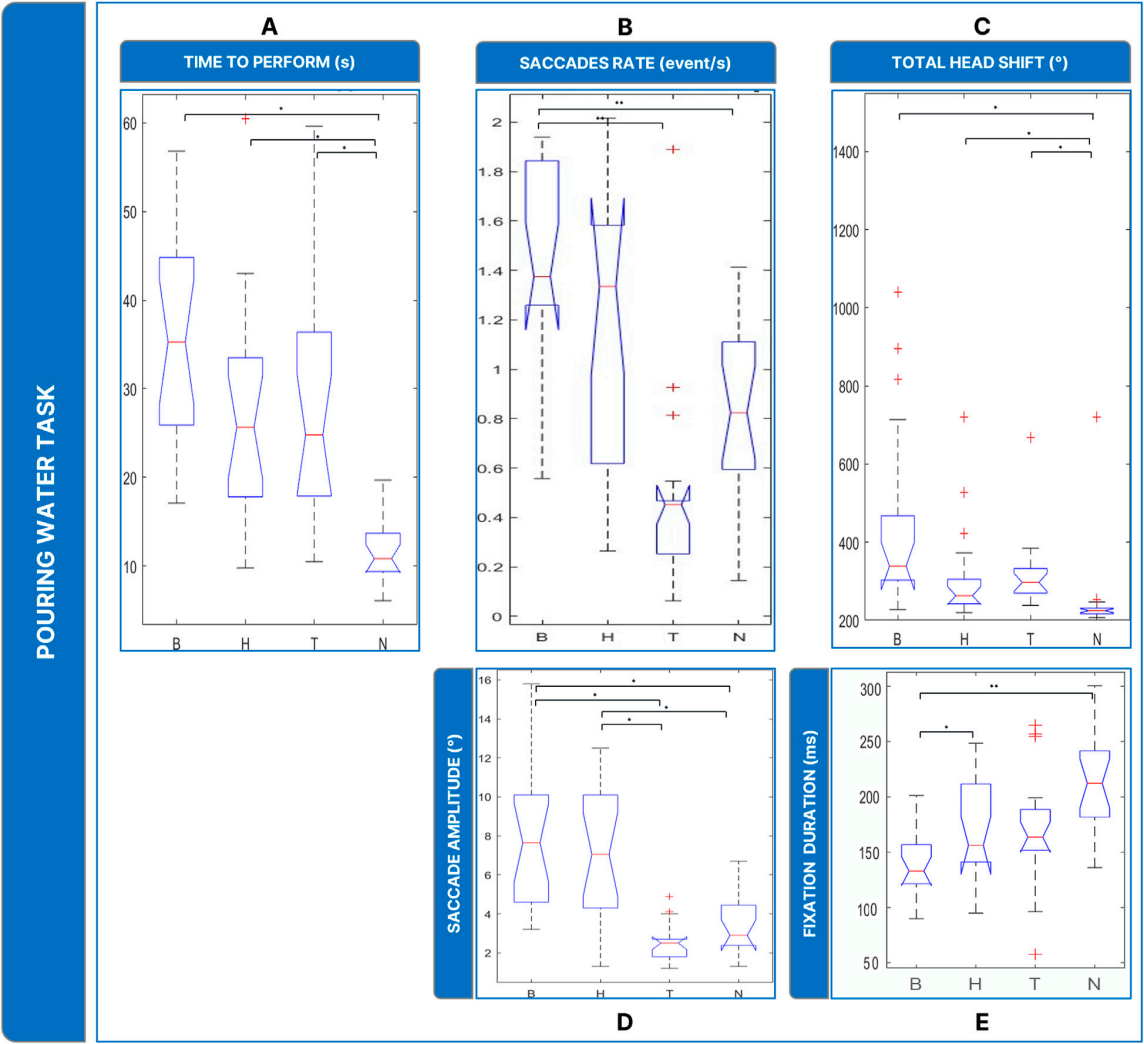
Boxplots of the results of the Pouring Water Task: Panel **(A)** time employed for completing the task; Panel **(B)** saccades rate; Panel **(C)** total head movements. Panel **(D)** saccades magnitude. Panel **(E)** fixations durations. Legend: (*: *p*-value ≤0.050).

Task performance was evaluated by counting the time spent performing the task (Figure 7A). Friedman’s Anova revealed significant differences between conditions ( $X^{2}$ = 33.7, *p* < 0.001). Post hoc testing revealed that, in terms of time spent to perform the task, low vision conditions required comparable time to perform the task, but there were significant differences from N (B vs. N: *p* < 0.001; H vs. N: *p* < 0.001; T vs. N: *p* = 0 .001).

The analysis of saccades rate ( $X^{2}$ = 22.7, *p* < 0.001) (Figure 7B) revealed that B showed a significantly higher rate of saccades than N (*p* = 0 .001), and T (*p* = 0 .010).

In addition, the amplitude of saccades (Figure 7D) ( $X^{2}$ = 28.1, *p* < 0.001) was larger wider in the low-vision conditions: B and H showed comparable saccades (∼3°, B vs. H: *p* = 1.000) and larger than T and N (∼7°, T vs. N: *p* = 0.313; T vs. B: *p* < 0.001; H vs. T: *p* = 0.003; B vs. N: *p* = 0.005; H vs. N: *p* = 0.021). In terms of fixation duration (Figure 7E), The Friedmann Anova revealed significant differences between conditions ( $X^{2}$ = 27.9, *p* < 0.001). Post hoc tests showed a significatively inferior value of fixation duration of B to N (*p* = 0.001) and to H (*p* < 0.010).

Finally, head rotations (Figure 7C) were more present in the low vision conditions ( $X^{2}$ = 30.9, *p* < 0.001): post-hoc tests revealed that B, H, and T induced the user to rotate the head more than in N (B vs. N: *p* < 0.001; T vs. N: *p* = 0.009, H vs. N: *p* < 0.001), while the behavior between the low vision conditions was comparable (H vs. B: *p* = 0.072; H vs. T: *p* = 1.000; B vs. T: *p* = 0.360).

### 3.4 Block insertion task

**FIGURE 8:**
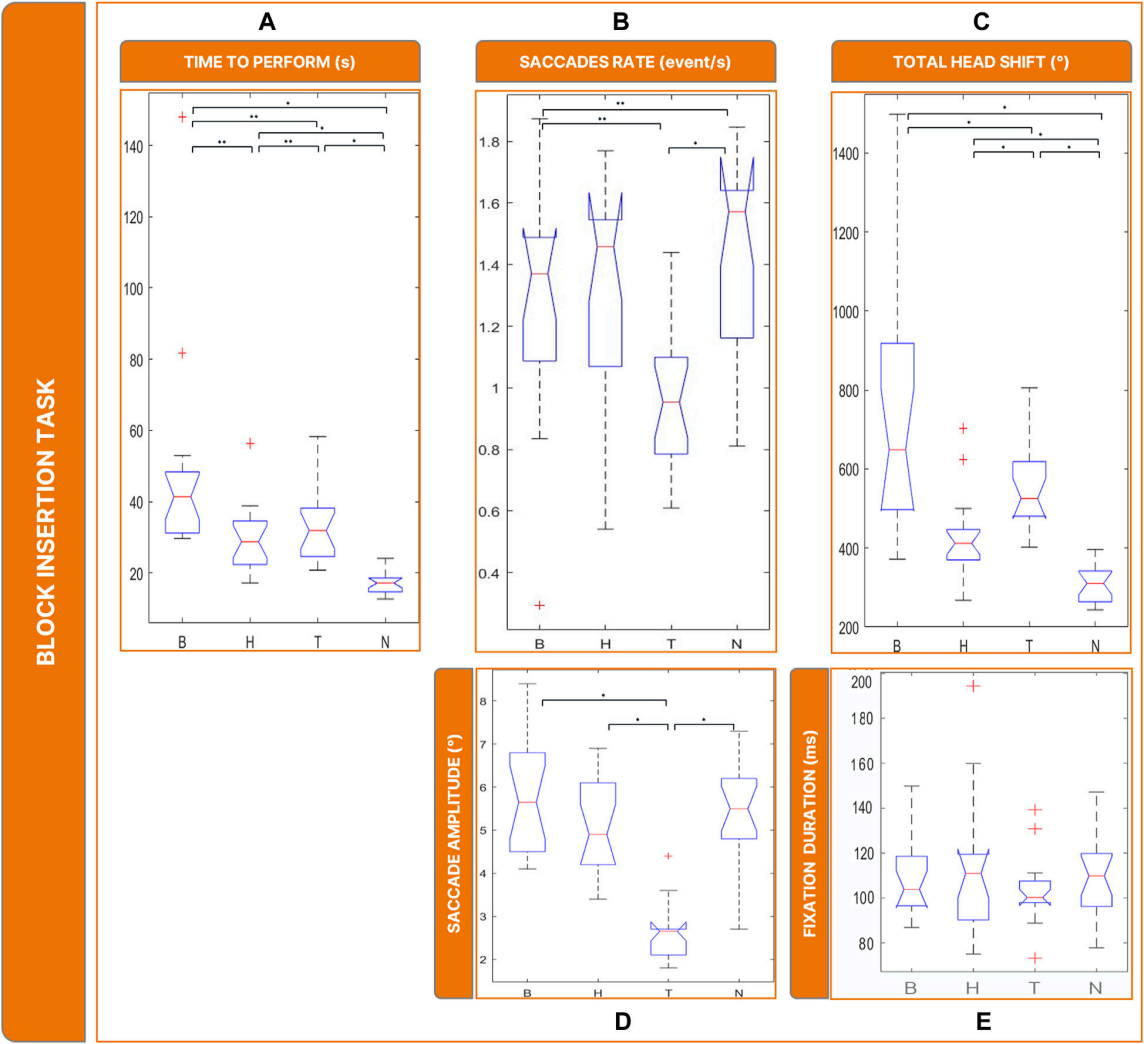
Boxplots of the results of the Block Insertion Task: Panel **(A)** time employed for completing the task; Panel **(B)** saccades rate; Panel **(C)** total head movements. Panel **(D)** saccades magnitude. Panel **(E)** fixations durations. Legend: (*: *p*-value <= 0.050).

Given that, as in the previous task, the required focus was on the central FOV, we reasonably found similarities with the water task. Counting the time spent performing the task (Figure 8A), a Friedman’s Anova revealed significant differences between conditions ( $X^{2}$ = 39.8, *p* < 0.001). Post-hoc tests revealed that all low vision conditions required more time than N to complete the task (B vs. N: *p* < 0 .001; H vs. N: *p* = 0.002; T vs. N: *p* < 0.001). In addition, B required more time to perform the task than H and T (B vs. H: *p* = 0.002; B vs. T: *p* = 0.020), while T and H were comparable (T vs. H: *p* = 0.979).

The rate of saccades (Figure 8B) was significantly different between conditions ( $X^{2}$ = 22.5, *p* < 0.001): specifically, T showed a lower rate of saccades (and higher rate of fixations) compared to all the other conditions (T vs. N: *p* = 0.001; T vs. B: *p* = 0.024; H vs. T: *p* = 0.002).

Saccade sizes (Figure 8D) were significantly different between conditions ( $X^{2}$ = 35.1, *p* < 0.001). In particular, post-hoc tests revealed that it was smaller in T than in all other conditions ∼2°, T vs. B: *p* = 0.002; T vs. H: *p* = 0.001; T vs. N: *p* = < 0.001). There is no significant difference in fixation duration ( $X^{2}$ = 1.130, *p* < 0.769) (Figure 8E).

The amount of head rotations (Figure 8C) differed significantly between conditions ( $X^{2}$ = 43.3, *p* < 0.001). Post-hoc tests revealed that the total rotation of the head was not significantly different in B and T (T vs. B: *p* = 0.360) and greater than in H (H vs. B: *p* < 0.001; H vs. T: *p* = 0.001). Additionally, all low vision conditions led the subject to move more than in N (B vs. N: *p* = < 0.001; T vs. N: *p* = < 0.001; H vs. N: *p* < 0.001).

## 4 Discussion

The first significant aspect explored in this study is the occurrence and characteristics of saccades and fixations in healthy-sighted subjects, particularly in relation to our healthy sight condition. Each ocular movement possesses distinct properties. For instance, saccades typically exhibit a specific range of amplitudes (4°–20°), while fixations tend to occur within durations ranging from 200 ms to 400 ms (Holmqvist et al., 2011a). Upon analyzing data obtained from subjects in the healthy sight condition (N), we observed that saccades demonstrated characteristics in terms of magnitude consistent with findings in the literature (Section 2.1). This observation was instrumental in confirming that the immersive system did not disrupt the execution process of saccades under healthy sight conditions, thereby facilitating further examination of data related to simulated low-vision conditions. On the other hand, in both reading and insertion bock tasks, fixation duration appeared to be under the average (Section 2.1).

We assessed the performance and oculomotor behavior of subjects across three different tasks simulating everyday activities (Section 3.1). Data pertaining to subjects’ behavior in simulated low-vision conditions, including eye and head movements, also exhibited similarities with alterations previously investigated in various studies (Goodwin, 2014; Nguyen et al., 2014; Verghese et al., 2021; McDonald et al., 2022) involving individuals affected by low-vision conditions (Section 3.2).

### 4.1 Evaluating subjects’ performance

In our evaluation of the reading task, we focused on the number of words read within a specific timeframe. It became evident that binocular maculopathy was the most debilitating low-vision condition in terms of task performance duration. Compared to homonymous hemianopsia and tunnel vision, the loss of central visual field significantly hindered reading ability. Given the task’s requirement for interaction within the central visual field, the presence of central scotomas naturally posed greater obstacles for subjects than in the healthy sight condition. The water pouring and block insertion tasks were assessed based on the time taken to complete them, as these tasks did not impose strict time constraints like the reading task. In both cases, subjects with low vision experienced similar impairments. Unlike the reading task, binocular maculopathy did not prove to be more debilitating than other low-vision conditions. We can speculate that the ability to rely on tactile senses and the occurrence of multisensory integration mitigated the impairments associated with binocular maculopathy. Indeed, Sathian (2000) reported that while sighted individuals primarily rely on vision, those with VIs rely more on tactile senses for pattern recognition. These findings are promising, as they suggest that individuals affected by homonymous hemianopsia and tunnel vision also exhibited slower performance in each task, similar to those who suffer from binocular maculopathy.

### 4.2 The impact of low-vision conditions on the oculomotor system and head movements

The presence of a central scotoma has been observed to induce instability in the oculomotor system (Nuthmann et al., 2022). Saccadic movements are also affected by the loss of central visual field, often resulting in irregular saccades and the splitting of linear saccades into smaller, irregular ones (Vullings and Verghese, 2021). Subjects affected by central scotomas tend to perform a significantly higher number of saccades with larger amplitudes compared to healthy-sighted subjects (Van der Stigchel et al., 2013). Our study confirmed these observations, particularly regarding the increased number of saccades and their wider amplitude in simulated binocular maculopathy compared to healthy sight conditions. However, unlike previous findings, the saccadic amplitude appeared wider rather than shorter, likely due to subjects’ strategy of using wide saccades to move the scotoma towards the peripheral visual field to obtain hidden information.

Subjects affected by homonymous hemianopsia exhibited similar oculomotor control instability as those with binocular maculopathy. Due to the essential information loss, individuals with hemianopsia perform multiple saccades to explore the environment (Grunda et al., 2013). In our study, simulated hemianopsia in the left hemifield resulted in diminished head scanning techniques compared to healthy sight conditions. Keeping the head still while performing saccades was the preferred strategy, aligning with previous findings (Goodwin, 2014).

Similarly, subjects affected by tunnel vision experience alterations in saccadic movements and fixations, with saccades being more numerous, shorter, and slower, and fixations being more unstable compared to healthy-sighted individuals (McDonald et al., 2022). In our study, simulated tunnel vision resulted in a small amount of shorter saccades across all tasks, particularly impacting tasks requiring object recognition, such as water pouring and block insertion.

In summary, subjects in healthy sight condition exhibited distinct behavior compared to those in low-vision conditions across all tasks. In simulated low-vision conditions, the higher rate of saccades indicated that scanning the environment with saccades was a natural technique to achieve task goals. Differences were also observed between binocular maculopathy and tunnel vision in terms of saccades and fixations, with compensatory strategies varying between conditions. The healthy sight condition prompted subjects to keep their heads more fixed compared to low-vision conditions, suggesting increased head movements as a strategy to succeed in tasks, particularly with simulated binocular maculopathy and tunnel vision.

These results suggest that altered reality is capable of inducing alterations in the oculomotor system of healthy-sighted individuals. From a qualitative perspective, we observed similarities in eye and head movements between participants and individuals affected by low vision conditions: multiple nonlinear saccades in binocular maculopathy; minimal head movement in homonymous hemianopsia; and shorter saccades in tubular vision. These findings motivate us to pursue further validation studies aimed at comparing clinical data from low-vision patients with those simulated using REALTER during the execution of our experimental protocol.

### 4.3 Future works and improvements

Our findings support the use of immersive XR gaze-contingent simulations as a valuable approach to enhance ophthalmologists’ training in understanding the effects of low vision conditions. REALTER can be utilized in research centers to investigate how VIs influence sensory perception skills, offering a novel perspective compared to studies involving blindfolded sighted individuals. Specifically, collaborating with professional ophthalmologists in further testing could provide valuable insights into compensatory techniques currently utilized in low-vision rehabilitation and aid in developing new techniques for individuals affected by maculopathy, tunnel vision, and hemianopsia.

In the future, validating the system by measuring the eye movements of low-vision individuals and comparing them with data obtained from healthy-sighted subjects in the simulated condition could aid in demonstrating the effectiveness and clinical relevance of the system.

Further experiments could provide valuable additional insights into understanding the behavioral alterations caused by low-vision conditions. For instance, conducting reading tests using standardized materials such as MNREAD Acuity Charts could offer an opportunity to collect data that could aid low-vision rehabilitators in devising novel rehabilitation techniques. Additionally, investigating the impact of tunnel vision on spatial navigation and visual searching during tasks involving free movement in space could be enlightening.

Given the limited sample size in related studies conducted on healthy individuals in simulated low-vision conditions, future tests should aim to include a larger number of participants. Moreover, due to the variability among healthy subjects in terms of saccadic and fixation properties and task performance, incorporating proper training sessions before experiments to familiarize subjects with simulated diseases could be beneficial. This approach could help determine subjects’ preferred retinal locus and shed light on how training affects task execution techniques.

Regarding the limitations of the immersive system, improvements could be made in terms of hardware, such as replacing the HTC Vive Pro Eye with a standalone HMD equipped with pass-through cameras and a more accurate eye tracker capable of recording ocular movements at a higher sampling frequency. Attention should be given to addressing issues related to gaze position accuracy and the inability to directly set the real-world position of the user’s eyes, which can impact the realism and effectiveness of simulations.

Considering the potential of immersive technology to simulate low vision, exploring the possibility of designing altered reality that simulates improvements for individuals with low vision could represent a significant advancement in rehabilitation. Despite challenges such as calibration issues, this perspective holds promise for enhancing low vision rehabilitation through immersive technology.

## 5 Conclusion

The primary focus of this study was to provide a quantitative assessment of the effectiveness of TR as an immersive low-vision simulator in inducing alterations in the oculomotor system and head movements in healthy-sighted individuals. The results obtained from the experiments demonstrated that: *i*) TR successfully reproduces everyday difficulties encountered by individuals affected by maculopathy, hemianopsia, and tubular vision in healthy-sighted individuals; *ii*) The TR simulator induces oculomotor system alterations in healthy-sighted individuals similar to those observed in the low vision disorders examined in this study.

Our simulator offers a comprehensive portfolio of low vision conditions compared to other studies (Chow-Wing-Bom et al., 2020; Jones et al., 2020), which primarily focused on glaucoma alone. By considering multiple low vision conditions such as maculopathy, hemianopsia, and tubular vision, we provided a more comprehensive analysis of the simulator’s capabilities in eliciting everyday difficulties experienced by individuals with various low vision conditions. Additionally, our preliminary analysis of eye and head movements revealed similarities between healthy-sighted subjects in simulated low vision and real individuals affected by VIs. This suggests that TR could be effectively utilized in rehabilitation facilities to train ophthalmologists in low-vision conditions and to study compensatory strategies, facilitating the development of new rehabilitation techniques.

In conclusion, the experiment highlighted significant characteristics unique to each simulated low vision condition. Given its reliability in assessing eye movement data, TR transcends its role as a mere training tool and serves as a valuable resource for advancing understanding of behavioral modifications and compensatory strategies in individuals with low vision. The system is primarily designed to aid ophthalmologists in understanding the limitations faced by patients with low vision, with the goal of developing tailored rehabilitation protocols and providing proper assistance to patients.

While caution should be exercised in interpreting results obtained from healthy subjects regarding VIs, we believe that our findings justify the integration of the simulator into research facilities to study low-vision individuals by conducting studies on healthy-sighted individuals. TR can be utilized in research centers to investigate the effects of low vision on sensory perception through the analysis of eye movements and head behavior.

In summary, TR holds promise for implementation in visual rehabilitation facilities to address the growing need for qualified rehabilitation professionals and to facilitate the development of user-centered and transdisciplinary approaches. Additionally, it serves as a valuable investigation tool for gaining deeper insights into low vision conditions in research institutes.

## Data Availability

The raw data supporting the conclusion of this article will be made available by the authors, without undue reservation.
